# Evidence of a Possible Viral Host Switch Event in an Avipoxvirus Isolated from an Endangered Northern Royal Albatross (*Diomedea sanfordi*)

**DOI:** 10.3390/v14020302

**Published:** 2022-02-01

**Authors:** Subir Sarker, Timothy R. Bowden, David B. Boyle

**Affiliations:** 1 Department of Microbiology, Anatomy, Physiology and Pharmacology, School of Agriculture, Biomedicine and Environment, La Trobe University, Melbourne, VIC 3086, Australia; 2 CSIRO Livestock Industries, Australian Animal Health Laboratory, Geelong, VIC 3220, Australia; timothy.bowden@csiro.au (T.R.B.); davidboyle48@gmail.com (D.B.B.); 3 CSIRO Australian Animal Health Laboratory, Australian Centre for Disease Preparedness, Geelong, VIC 3220, Australia

**Keywords:** avipoxvirus, host-switch, evolution, northern royal albatross

## Abstract

Avipoxviruses have been characterized from many avian species. Two recent studies have reported avipoxvirus-like viruses with varying pathogenicity in reptiles. Avipoxviruses are considered to be restricted to avian hosts. However, reports of avipoxvirus-like viruses from reptiles such as the green sea turtle (*Chelonia mydas*) and crocodile tegu (*Crocodilurus amazonicus*) suggest that cross-species transmission, within avian species and beyond, may be possible. Here we report evidence for a possible host switching event with a fowlpox-like virus recovered from an endangered northern royal albatross (*Diomodea sanfordi*)—a species of *Procellariiformes*, unrelated to *Galliformes*, not previously known to have been infected with fowlpox-like viruses. Complete genome sequencing of this virus, tentatively designated albatrosspox virus 2 (ALPV2), contained many fowlpox virus-like genes, but also 63 unique genes that are not reported in any other poxvirus. The ALPV2 genome contained 296 predicted genes homologous to different avipoxviruses, 260 of which were homologous to an American strain of fowlpox virus (FWPV). Subsequent phylogenetic analyses indicate that ALPV2 likely originated from a fowlpox virus-like progenitor. These findings highlight the importance of host-switching events where viruses cross species barriers with the risk of disease in close and distantly related host populations.

## 1. Introduction

Over the past several decades, marine bird populations have been decreasing globally [1] with the sustainability of the albatrosses (family *Diomedeidae*) and large petrels (*Macronectes* and *Procellaria* spp.) being under significant threat [2,3,4]. One of the world’s largest seabirds, the northern royal albatross (*Diomedea sanfordi*) is categorized as an “endangered” species under the International Union for Conservation of Nature (IUCN) Red List and is classified as Category B for management urgency [5]. Northern royal albatrosses range extensively throughout the Southern Ocean, yet infrequently into Antarctic waters. Breeding colonies are confined to the Chatham Islands and Taiaroa Head on the Otago Peninsula, Dunedin, New Zealand. The overall breeding population in the Chatham Islands colonies (99% of the total) is anticipated at about 6500–7000 pairs, which equates to a total population of approximately 17,000 adults [6].

Human activities, including commercial fishing and pollution, are recognized as threats for incidental mortality of these species [5,7,8,9,10,11]. Invasive alien species, degradation or destruction of nesting grounds, storms and flooding, pollution of the marine environment, and ingestion of plastics are additional factors contributing to population declines [5,12]. Infectious diseases, including those caused by avipoxviruses, are also recognized as potential threats to the preservation of small and endangered bird populations, including albatrosses [13,14,15,16,17,18]. Population declines in multiple species from the avifauna of Hawaii have been attributed to the introduction of avipoxviruses [19]. Avipoxviruses have also been identified as an ongoing threat to birds on the Galapagos Islands [20]. In contrast, despite the severity of avipoxvirus infections in some chicks, the high recovery rate, fledgling success, and post-fledgling survival observed in Hawaiian Laysan albatrosses (*Phoebastria immutabilis*), even in years of high rainfall when the prevalence of infections in chicks averaged as high as 88%, suggested that this particular albatross species has good immunity to avian poxviruses [21].

Avipoxviruses are large, double-stranded DNA (dsDNA) viruses belonging to the genus *Avipoxvirus* in the subfamily *Chordopoxvirinae*, family *Poxviridae*. Avipoxviruses have been detected in at least 374 avian species from 23 orders of wild and domestic avifauna worldwide [22,23,24,25]. Strikingly, recent studies have discovered two avipoxvirus-like viruses of varying pathogenicity in reptiles [26,27]. Sarker et al. [26] reported the presence of a novel avipoxvirus-like virus, cheloniid poxvirus 1 (ChePV-1), from an endangered green sea turtle (*Chelonia mydas*). ChePV-1, characterized microscopically and genetically from a cutaneous lesion, was shown to be considerably different from other known poxviruses but showed the greatest sequence identity (89.3%) to an avipoxvirus (shearwater poxvirus 2 (SWPV2)). Another recent study characterized a novel poxvirus, teiidaepox virus 1 (TePV-1), in cutaneous tissue of a lizard species, crocodile tegu (*Crocodilurus amazonicus*), native to the Amazon and Orinoco basins of South America [27]. TePV-1 was also closely related genetically and phylogenetically to avipoxviruses, and showed the highest amino acid sequence identities to flamingopox virus (74.3%) and fowlpox virus (76.0%) using the DNA polymerase and DNA topoisomerase genes, respectively [27]. The authors highlighted a possible virus transmission event between avian and reptilian species [27]. In addition, evidence of an avipoxvirus infection was reported in a critically ill rhinoceros in 1969, and this was characterized as an atypical fowlpox virus [28], although genome sequence data of the virus have not been reported.

Herein we report the genomic characterization of a second novel avipoxvirus, ALPV2, isolated from a skin lesion collected from an endangered northern royal albatross on the Otago Peninsula, New Zealand. In contrast to the previously reported ALPV isolate from this region [29], and considering that the northern royal albatross (*Diomedea sanfordi*) is a species of *Procellariiformes* unrelated to *Galliformes*, we provide evidence that ALPV2 represents a possible host switch event with a fowlpox virus-like progenitor.

## 2. Materials and Methods

### 2.1. Sampling, Virus Isolation and Genome Sequencing

Cutaneous pox lesions were collected from an endangered juvenile northern royal albatross (*Diomedea sanfordi*), located on the Otago Peninsula, near Dunedin, on the South Island of New Zealand. Sampling was conducted in March 1997 by Wallaceville Animal Research Centre, New Zealand, and lesions were sent to the Australian Animal Health Laboratory, Geelong, Victoria, Australia (sample ID: TST 08/05/1997). Virus isolation and propagation, as well as DNA extraction and genome sequencing, were subsequently undertaken as described previously [29].

### 2.2. Genome Assembly and Annotation

The resulting 3,374,936 paired-end raw sequence reads were used to assemble the complete genome of ALPV2, using CLC Genomics Workbench (version 9.5.4, CLC bio, a QIAGEN Company, Prismet, Aarhus C, Denmark) and Geneious (version 10.2.2, Biomatters, New Zealand), as described previously [29,30,31,32,33]. This resulted in the generation of a 286,155 bp genome. Clean raw reads (2.38 million) were mapped back to the assembled ALPV2 genome resulting in an average coverage of 668.55x. The genome was annotated according to the previously published protocol [18] using Geneious software (version 10.2.2, Biomatters, New Zealand). Open reading frames (ORFs) longer than 30 amino acids, with a methionine start codon (ATG) and minimal overlap with other ORFs (not exceeding 50% of one of the genes), were selected and annotated. ORFs shorter than 30 amino acids that had been previously annotated in other poxvirus genomes were also included. Similarity BLAST searches were performed on the predicted ORFs and were annotated as potential genes if predicted ORFs showed significant sequence similarity to known viral or cellular genes (BLAST E value ≤ e^−5^) [34].

To predict the function of putative unique ORFs identified in this study, the derived protein sequence of each ORF was searched using multiple applications to identify conserved domains or motifs. Transmembrane helices were searched using the TMHMM package (version 2.0) [35] and TMpred [36]. Additionally, searches for conserved secondary structure (HHpred) [37] and protein homologs were conducted using Phyre2 [38] and SWISS-MODEL [39].

### 2.3. Comparative Genomics

Genomic features of the newly sequenced ALPV2 were visualised using Geneious (version 10.2.2). Sequence similarity percentages between representative Chordopoxvirus (ChPV) and ALPV2 complete genome sequences were determined using tools available in Geneious (version 10.2.2). Dot plots were created based on the EMBOSS dottup program in Geneious software, with word size = 12 [40].

### 2.4. Phylogenetic Analyses

Phylogenetic analyses were performed using the ALPV2 genome sequence determined in this study, together with other selected ChPV genome sequences available in GenBank (Table 1). Nucleotide sequences of the partial DNA polymerase and partial p4b genes, as well as concatenated amino acid sequences of nine poxvirus core proteins, were aligned as described previously [41] using the MAFTT L-INS-I algorithm implemented in Geneious (version 7.388) [42]. To determine the best-fit model to construct phylogenetic analyses, a model test was performed using CLC Genomics Workbench (version 9.5.4), which favoured a general-time-reversible model with gamma distribution rate variation and a proportion of invariable sites (GTR+G+I). Phylogenetic analyses for nucleotide sequences were performed under the GTR substitution model, but the WAG substitution model was chosen for concatenated amino acid sequences with 1000 bootstrap replicates in CLC Genomic Workbench (version 9.5.4).

## 3. Results

### 3.1. Genome of Albatrosspox Virus 2 (ALPV2)

We determined the complete genome sequence of ALPV2 as a linear double-stranded DNA molecule 286,155 bp in length (GenBank OK348853). The ALPV2 genome encompassed a large central coding region surrounded by two matching inverted terminal repeat (ITR) regions, constituting 7781 bp each (coordinates 1-7781 sense and 278,375–298,392 antisense orientation) similar to other characterized avipoxviruses [30,31,32,41,45,51]. The ALPV2 genome showed the highest nucleotide identity (99.3%) with an American virulent strain of fowlpox virus isolated in 1999 (GenBank accession no. AF198100.1) [45] (Table 2), followed by PEPV (76.6%), FGPV (75.0%), and FeP2 (73.1%). However, the ALPV2 genome showed much lower nucleotide identity (48.8%) with the only other reported albatrosspox virus (ALPV), which had also been collected in March 1997 from a northern royal albatross on the Otago Peninsula, New Zealand [29]. The A+T content of the ALPV2 genome was 69.1%, which was comparable to other sequenced avipoxviruses (Table 2).

### 3.2. Genome Annotation and Comparative Analyses of ALPV2

**Figure 1:**
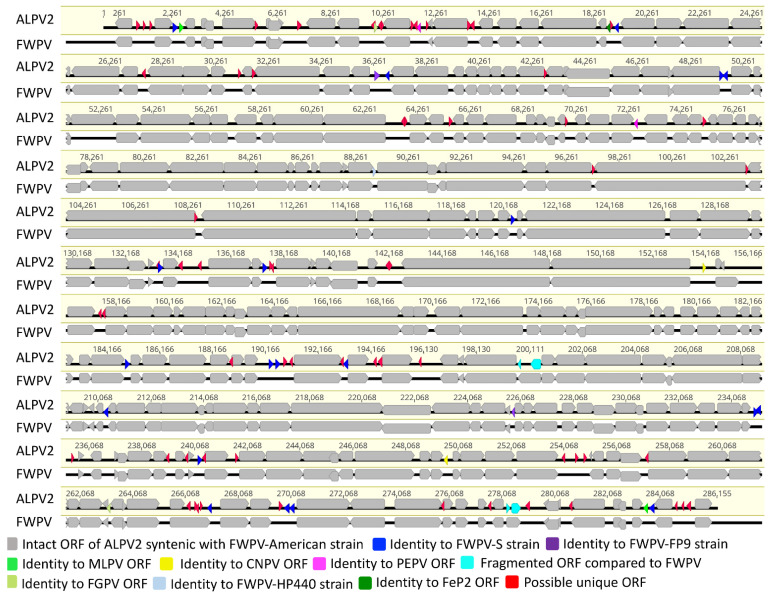
Genomic illustration of ALPV2. The arrows depict the direction of transcription of genes and open reading frames (ORFs). Each gene or ORF is color coded, as indicated by the key in the legend.

The ALPV2 genome contained 359 predicted open reading frames (ORFs) encoding proteins ranging from 30 to 1918 amino acids in length. Putative genes were numbered from left to right (Figure 1 and Supplementary Table S1). Among them, 16 predicted ORFs were located within the ITRs and were thus present as diploid copies. Comparative analysis of the predicted ORF sequences was conducted, and 298 of the ORFs had greatest similarity with other ChPV gene products (E value ≤ 10^−5^) (Figure 1 and Supplementary Table S1). Among these predicted genes, 260 showed the highest similarity to an American strain of fowlpox virus (FWPV) [45]. A further twenty (ORF-006, -039, -57, -74, -75, -144, -154, -160, -208, -216, -217, -222, -244, -274, -275, -287, -327, -331, -332 and -354) showed highest similarity to fowlpox virus vaccine strain (FWPV-S) [48], two (ORF-056 and -260) to FWPV strain FP9 [55], one (ORF-125) to FWPV strain HP440 [56], two (ORF-007 and -353) to MLPV [41], two (ORF-020, -318) to FGPV [24], two (ORF-026 and -103) to PEPV [51], two (ORF-172 and- 299) to CNPV [43] and one (ORF-037) to FeP2 [51] (Figure 1 and Supplementary Table S1). In comparison to FWPV, none of the genes were absent in the ALPV2 genome, and a further four ORFs (ORF-231, -232, 342 and -343) were truncated and or fragmented (Figure 1 and Supplementary Table S1). Two fragmented ORFs of ALPV2 (ORF-231 and -232) are conserved chordopoxvirus genes, whereas the other two (ORF-342 and -343) are non-conserved genes. Additional studies will be needed to determine whether fragmented ORFs are expressed and functional.

Remarkably, ALPV2 contained 63 predicted protein-coding genes that were not present in any other poxvirus, nor did they match any sequences in the NR protein database using BLASTX and BLASTP [34]. These unique ORFs’ encoded proteins of 30 to 48 amino acids in length (Figure 1 and Supplementary Table S1). Among them, 21 unique ALPV2 protein-coding ORFs (ORF-016, -019, -030, -050, -052, -066, -088, -107, -132, -156, -157, -162, -213, -219, -224, -225, -288, -311, -323, -340, -344) were predicted to contain a single transmembrane helix, and a further two (ORF-024 and -031) were predicted to contain two transmembrane helices using the software packages employed in this study (Supplementary Table S1). However, we did not find any significant homology with known proteins for the unique ORFs encoded in the ALPV2 genome when using the Phyre2, HHpred and SWISS-MODEL, which might be due to the lack of closely related structures in these databases.

**Figure 2:**
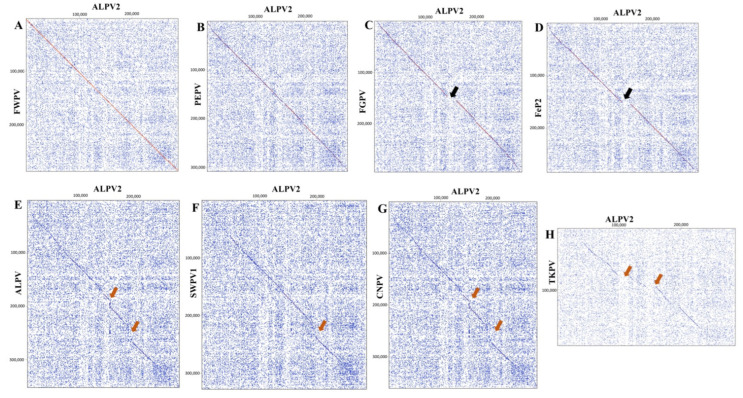
Dot plots of the ALPV2 genome (*x*-axis) versus other poxvirus genomes (*y*-axis). (**A**) ALPV2 vs. FWPV; (**B**) ALPV2 vs. PEPV; (**C**) ALPV2 vs. FGPV; (**D**) ALPV2 vs. FeP2; (**E**) ALPV2 vs. ALPV; (**F**) ALPV2 vs. SWPV1; (**G**) ALPV2 vs. CNPV; and (**H**) ALPV2 vs. TKPV (refer to Table 1 for virus details and GenBank accession numbers). The Classic color scheme was chosen in Geneious (version 10.2.2) for the dot plot lines according to the length of the match, from blue for short matches to red for matches over 100 bp long. Window size = 12.

Comparison of the ALPV2 genome with those of other avipoxviruses was performed using dot plot analyses. The ALPV2 genome was highly syntenic with FWPV, PEPV, FGPV and FeP2 (Figure 2A–D). Moreover, differences in synteny with FGPV and FeP2 were observed (Figure 2C–D, highlighted as black arrows), mainly due to the presence of two large additional copies of variola B22R gene family proteins in ALPV2 (ORF-170 and -171, 1870 and 1766 amino acids in length, respectively). The ALPV2 genome also demonstrated significant differences in the entire genome compared to ALPV, SWPV1, CNPV and TKPV (Figure 2E–H). Within these highlighted regions (orange arrows), multiple SNPs and insertions/deletions (indels) accounted for the variation observed between the genomes (Figure 2E–H).

### 3.3. Core/Conserved ORFs

Similarly to other ChPVs, the ALPV2 genome contained 87 conserved core genes, which are involved in essential functions such as replication, transcription and virion assembly (Supplementary Table S1; highlighted with bold and italic font). The number of conserved ChPV genes is considered to range between 83-90 [24,51,57,58], which is consistent with the findings in the ALPV2 genome. Among them, two of the predicted ORFs, encoding immunodominant virion proteins ALPV-231 and -232, were truncated compared to FWPV168, which may warrant further studies to determine whether they are expressed and functional. Based on a recent study by Carulei et al. [24], we also searched for a further 47 genes that are conserved in avipoxviruses (Table 3). The ALPV2 genome was also predicted to contain these 47 conserved ORFs (Table 3), and none of the genes were found to be truncated or fragmented compared to a closely related fowlpox virus (FWPV) [45].

### 3.4. Multigene Families

Avipoxviruses are the largest ChPVs and contain several, large, multigene families with immune related functions comprising up to 50% of the genome [24,51]. Table 4 shows the copy numbers of each of the 14 multigene families identified in the ALPV2 genome compared with the other selected sequenced avian poxvirus genomes, including the recently characterized genomes of ALPV, MPPV2 and PEPV2. ALPV2 has a similar complement of multigene families compared to FWPV (total of 95 and 89 gene copies, respectively). However, ALPV2 has a significantly lower number of multigene families in comparison with ALPV (GenBank accession no. MW365933.1) (total of 95 and 139 gene copies, respectively). The copy number of ankyrin repeat, N1R/p28, C-type lectin and TGF-β family genes were significantly higher in the ALPV2 genome compared to ALPV.

### 3.5. Evolutionary Relationships of ALPV2

**Figure 3:**
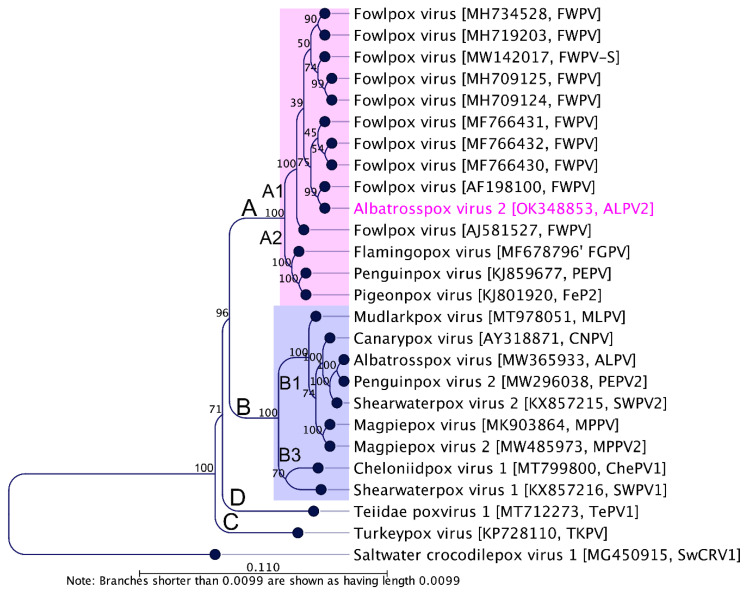
Phylogenetic relationships between ALPV2 and other chordopoxviruses. A maximum likelihood (ML) tree was constructed from multiple alignments of the concatenated amino acid sequences of the selected nine poxvirus core proteins using CLC Genomics Workbench (version 9.5.4). The numbers on the left show bootstrap values as percentages. The labels at branch tips refer to virus species, followed by GenBank accession numbers and abbreviated species names in parentheses. The position of ALPV2 is highlighted using pink text. Details of the poxviruses used in the phylogenetic tree are in Table 1. Saltwater crocodile poxvirus 1 (SwCRV1; MG450915) [53] was used as an outgroup. Major clades and sub-clades are designated according to Gyuranecz et al. (2013) [59].

**Figure 4:**
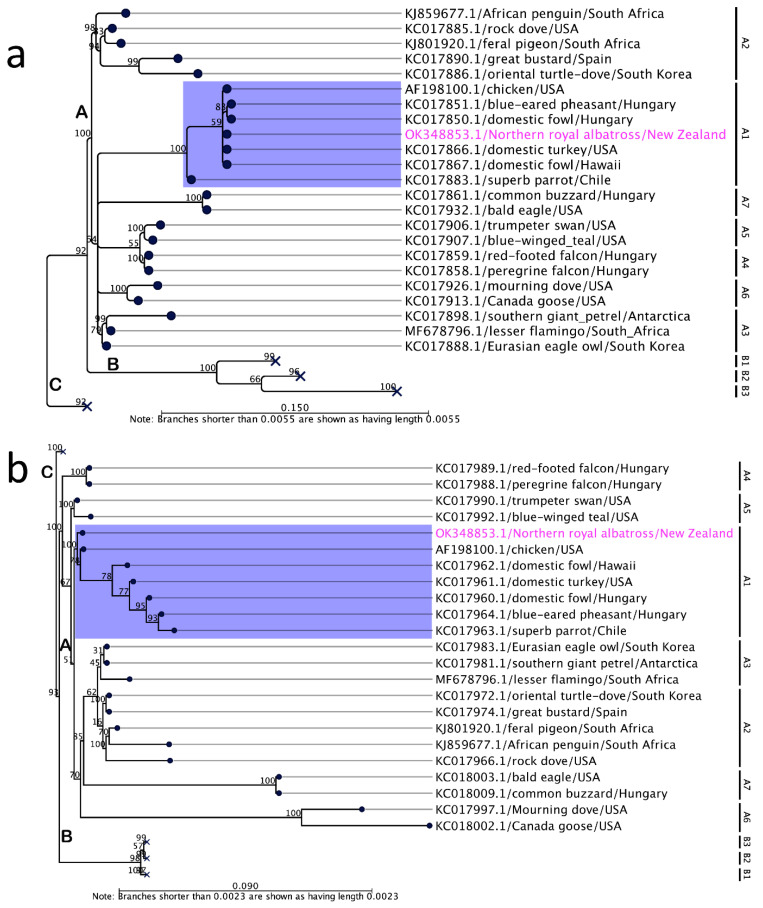
Maximum likelihood (ML) phylogenetic tree from partial nucleotide sequences of the DNA polymerase gene (**a**) and P4b gene (**b**) of selected avipoxviruses. Labels at branch tips refer to GenBank accession number/species/country of origin. The numbers on the left show bootstrap values as percentages. The relevant sub-clade A1 is highlighted using blue shading, whilst the position of ALPV2 is highlighted using pink text. The ML tree is displayed as a phylogram. The bootstrap value assigned to a node in the output tree is the percentage (0–100) of the bootstrap resamples which resulted in a tree containing the same subtree as that rooted at the node. Major clades and sub-clades are designated according to Gyuranecz et al. (2013) [59]. Major clades B and C in both the trees (**a**) and (**b**) are collapsed. For the complete ML phylograms, please see Supplementary Figures S1 and S2.

Phylogenetic reconstruction using concatenated amino acid sequences of selected conserved ChPV genes provides clear evidence for the inclusion of ALPV2 in the genus *Avipoxvirus.* In the maximum likelihood (ML) tree (Figure 3), ALPV2 was located within a sub-clade A1 encompassing mostly FWPV isolates from chickens and wild turkeys with 100% bootstrap support. Using the same set of concatenated protein sequences, we found that the maximum inter-lineage sequence identity values ranged from 99% to 100% among FWPV isolates from chickens and turkeys, and ALPV2, respectively, which illustrated the phylogenetic position of this avipoxvirus sequenced from an endangered northern royal albatross, and further inferred that these viruses were likely derived from a common progenitor. Strikingly, a few other avipoxviruses were also positioned in the same A1 sub-clade when we used partial nucleotide sequences of the DNA polymerase and p4b genes (Figure 4). This included poxviruses isolated from birds of the order *Galliformes* (domestic fowl, *Gallus domesticus*; blue-eared pheasant, *Crossoptilon auritum*) in Hungary and Hawaii, respectively [59], and a *Psittaciformes* (superb parrot, *Polytelis swainsonii*) originating from Chile [59], which are almost identical to ALPV2 within this relatively small fragment of the genome.

## 4. Discussion

We have documented a second avipoxvirus infection in a northern royal albatross, notably involving a possible host switch event. Applying various approaches for genomic comparison, ALPV2 was shown to be genetically similar to other avipoxviruses with the highest nucleotide sequence similarity being to an American virulent strain of fowlpox virus (99.3%), followed by PEPV (76.6%), FGPV (75.0%), and FeP2 (73.1%). The AT content of the ALPV2 genome was 69.1%, which was consistent with previously reported avipoxviruses. Given the phylogenetic relationship of ALPV2 (Figure 3 and Figure 4; Supplementary Figures S1 and S2), we propose that this virus, in contrast to ALPV [29], originated from a common ancestor that deviated from a fowlpox virus-like progenitor. Notably, there were significant differences observed between the ALPV2 and ALPV genomes (regarding nucleotide identity, genome length and number of ORFs (Table 2)), confirming that two distinct avipoxviruses were present in juvenile northern royal albatrosses sampled on the Otago Peninsula (South Island of New Zealand) in March 1997.

Interestingly, the phylogenetic relationship of ALPV2 using concatenated protein sequences encoded by the chosen poxvirus core genes (Figure 3), and partial nucleotide sequences of DNA polymerase and P4b genes (Figure 4), demonstrate that the newly assembled albatrosspox virus genome is representative of a sub-clade A1 avipoxvirus. In support of our findings, nearly identical partial nucleotide sequences of fowlpox viruses were isolated from other non-chicken avian species including blue-eared pheasant (*Crossoptilon auritum*) in Hawaii [59] and a *Psittaciformes* (superb parrot, *Polytelis swainsonii*) originating from Chile [59], which are also positioned in the same A1 sub-clade. In addition, an atypical fowlpox virus was also reported from a fatally ill rhinoceros in 1969 [28]; however, genetic characterization of this virus has not been reported. Two recent studies of avipoxvirus-like viruses provide examples of viruses able to infect non-avian hosts: cheloniid poxvirus 1 (ChePV-1) and teiidaepox virus 1 (TePV-1), which have been linked with infections in green sea turtle and crocodile tegu [26,27]. Our findings suggest an unusual transmission event may have occurred, resulting in lesions, representing either a “dead-end” event and/or that fowlpox-like viruses may have a wider host range with the ability to cross chicken-host barriers. Characterization of additional avipoxviruses isolated from these endangered albatross species may enable the likelihood and importance of cross species transmission of fowlpox and fowlpox-like viruses to be determined.

It is not possible to explain the host–pathogen dynamics of ALPV2 from this case alone, but it is apparent that northern royal albatrosses and other avian species share an ecological habitat. Northern royal albatrosses are typically solitary foragers, however they may gather at food sources at sea. Most of their food is thought to be obtained by feeding on dead or dying prey from the surface and by scavanging fish, rubbish and bait discarded from commercial fishing boats. This may indicate a possible scenario for virus transmission that requires further investigation. Another potential route of virus transmission is mechanical, where biting arthropods are assumed to play a part in the spread of avipoxviruses within wild bird populations. Ticks, fleas [60], hippoboscid flies [61], and mosquitos [19,62] may all play a role as mechanical vectors. Northern royal albatrosses also congregate at breeding colonies where transmission of avipoxviruses by insect vectors is likely to occur.

Avipoxviruses have been reported to infect other albatross species, including a critically endangered Waved Albatross (*Phoebastria irrorata*) [63] and Laysan Albatross (*Phoebastria immutabilis*) [21]. The severity of avipoxvirus infections is variable and infrequently fatal, although secondary bacterial and fungal infections may subsequently occur and cause mortality [23]. Nevertheless, the pathogenic effects of avipoxvirus infections in seabirds are not adequately understood, and the longer-term consequences in susceptible populations remain to be determined. The genetic information provided in this study will facilitate improved understanding of the evolution of poxviruses in this avian species, thus contributing to the development of improved management and conservation strategies for the endangered northern royal albatross.

## 5. Conclusions

We have reported the genomic characterization of a second novel avipoxvirus, tentatively designated ALPV2, isolated from an endangered northern royal albatross on the Otago Peninsula, New Zealand, and provide evidence that this infection resulted from a possible host switch event. The ALPV2 genome was similar to those of other avipoxviruses and likely originated from a common ancestor that deviated from a fowlpox virus-like progenitor. This finding has increased our knowledge of the pathogen diversity within northern royal albatrosses in New Zealand. However, additional investigations will be required to better understand relevant host–pathogen dynamics including routes of transmission and factors leading to infection, associated pathology and disease prevalence.

## Figures and Tables

**Table 1:** Related poxvirus genome sequences used in further analysis of ALPV2.

Virus	Abbreviation	Year of Isolation	GenBank Accession Number	Reference
Albatrosspox virus 2	ALPV2	1997	OK348853	This study
Albatrosspox virus	ALPV	1997	MW365933	[29]
Canarypox virus	CNPV	1948, 2015	AY318871, MG760432	[43,44]
Cheloniidpox virus 1	ChePV1	2018	MT799800	[26]
Fowlpox virus	FWPV	2012, 2000 *, 2010 *, 2015, 2016, 2018 *, 2011 ^#^, 2018, 2010 ^#^	MW142017, AF198100 *, AJ581527 *, MH734528, MH719203, MF766430-32, MH709124-25 *, MG702259 ^#^, OK558608-09, KX196452 ^#^	[45,46,47,48,49]
Flamingopox virus	FGPV	2008	MF678796	[24]
Magpiepox virus	MPPV	2018	MK903864	[32]
Magpiepox virus 2	MPPV2	1956	MW485973	[50]
Mudlarkpox virus	MLPV	2019	MT978051	[41]
Penguinpox virus	PEPV	1992	KJ859677	[51]
Penguinpox virus 2	PEPV2	1997	MW296038	[18]
Pigeonpox virus	FeP2	1992	KJ801920	[51]
Saltwater crocodilepox virus 1	SwCRV1	2017	MG450915	[52,53]
Shearwaterpox virus 1	SWPV1	2015	KX857216	[30]
Shearwaterpox virus 2	SWPV2	2015	KX857215	[30]
Turkeypox virus	TKPV	2011	NC_028238	[54]
Teiidae poxvirus 1	TePV-1	2019	MT712273	[27]

* = year of submission to GenBank; ^#^ = unpublished.

**Table 2:** Comparative analysis of representative avipoxviruses and ALPV2 based on complete genome nucleotide sequences.

Avipoxvirus (Abbreviation)	GenBank Accession Number	Genome Identity (%)	Genome Length (kbp)	A+T Content (%)	Number of ORFs	Reference
Albatrosspox virus 2 (ALPV2)	OK348853		286	69.1	359	This study
Albatrosspox virus (ALPV)	MW365933	48.8	352	71.2	336	[29]
Canarypox virus (CNPV)	AY318871	48.0	360	69.6	328	[43]
Fowlpox virus (FWPV)	AF198100	99.3	289	69.1	260	[45]
Flamingopox virus (FGPV)	MF678796	75.0	293	70.5	285	[24]
Magpiepox virus (MPPV)	MK903864	51.1	293	70.4	301	[32]
Magpiepox virus 2 (MPPV2)	MW485973	50.8	298	70.5	419	[50]
Mudlarkpox virus (MLPV)	MT978051	49.0	343	70.2	352	[41]
Penguinpox virus (PEPV)	KJ859677	76.6	307	70.5	285	[51]
Penguinpox virus 2 (PEPV2)	MW296038	49.0	350	69.9	327	[18]
Pigeonpox virus (FeP2)	KJ801920	73.1	282	70.5	271	[51]
Shearwaterpox virus 1 (SWPV1)	KX857216	52.2	327	72.4	310	[30]
Shearwaterpox virus 2 (SWPV2)	KX857215	48.4	351	69.8	312	[30]
Turkeypox virus (TKPV)	KP728110	40.3	189	70.2	171	[54]

**Table 3:** 47 ORFs found to be uniquely conserved in the selected fully sequenced avian poxvirus genomes.

ALPV2	FWPV	MPPV2	MPPV	SWPV2	SWPV1	CNPV	PEPV	FeP2	FGPV	TKPV	Function
33	16	44	34	28	24	32	19	19	11	001.1a	Ig-like domain
34	17	45	35	29	25	33	20	20	12	2	V-type Ig domain
40	20	53	41	34	28	38	24	24	17	5	C4L/C10L protein
41	21	54	42	35	29	39	25	25	18	6	GPCR
42	22	55	43	36	30	40	26	26	19	7	Ankyrin repeat
43	23	57	44	37	31	41	27	27	20	8	Ankyrin repeat
44	24	58	45	38	32	42	28	28	21	9	Ankyrin repeat
53	30	66	52	44	38	48	35	35	29	12	Alkaline phosphodiesterase
54	31	69	55	46	40	50	36	36	30	13	Ankyrin repeat
60	35	72	58	49	44	53	40	38	34	16	Hypothetical protein
62	37	74	60	51	46	55	41	39	36	17	Hypothetical protein
64	39	77	63	54	49	58	43	41	38	20	B-cell lymphoma 2 (Bcl-2)
65	40	78	64	55	50	59	44	42	39	21	Serpin
69	43	81	66	57	52	61	46	44	41	22	DNA ligase
70	44	82	67	58	53	62	47	45	42	23	Serpin family
71	46	83	68	59	54	63	48	46	43	24	Hydroxysteroid dehydrogenase
73	47	87	71	61	56	65	49	47	44	25	Semaphorin
76	48	92	75	64	59	68	50	48	45	26	GNS1/SUR4
82	54	103	83	72	66	76	56	54	51	32	mutT motif
96	65	-	-	83	78	88	67	65	64	40	Hypothetical protein
100	68	128	98	87	82	92	70	68	67	42	Hypothetical protein
102	70	130	100	89	84	94	72	70	69	44	T10-like protein
104	71	-	104	92	87	97	75	72	72	46	Hypothetical protein
109	75	140	110	98	92	103	78	77	76	50	N1R/p28
120	86	150	120	108	102	113	89	87	87	60	Thymidine kinase
126	91	156	126	113	107	118	95	93	93	65	Hypothetical protein
127	92	157	127	114	108	119	96	94	94	66	Hypothetical virion core protein
142	104	169	139	126	120	131	108	106	106	75	Hypothetical protein
143	105	170	140	127	121	132	109	107	107	76	Hypothetical protein
149	110	175	145	132	126	137	114	112	112	80	Hypothetical protein
152	113	178	148	135	129	140	117	115	115	83	Hypothetical protein
196	145	243	199	179	167	191	153	146	151	109	Hypothetical protein
203	151	253	209	187	175	199	159	153	157	113	Deoxycytidine kinase
255	190	331	274	250	237	264	203	195	204	140	A-type inclusion protein
256	191	333	275	251	238	265	204	196	205	141	A-type inclusion protein
262	196	339	280	256	243	270	210	202	211	144	Hypothetical protein
267	201	343	284	259	247	273	215	207	216	149	Hypothetical protein
269	203	344	285	260	248	274	216	208	217	150	Tyrosine kinase
271	205	346	287	262	250	276	218	210	219	151	Hypothetical protein
273	207	348	289	264	252	278	220	212	221	151.1a	Hypothetical protein
277	208	351	292	267	255	281	222	214	224	152	Hypothetical protein
280	211	355	296	271	259	285	225	216	227	153	Epidermal Growth Factor
281	212	356	297	272	260	286	226	217	228	154	Serine/threonine protein kinase
282	213	357	298	273	261	287	227	218	229	155	Hypothetical protein
284	214	361	300	275	263	289	228	219	230	156	Putative 13.7 kDa protein
293	219	370	308	282	272	296	234	226	238	161	Ankyrin repeat
312	232	394	327	290	283	304	248	238	251	164	Ankyrin repeat

Note: the numbers in each column refer to the specific ORF in each respective genome.

**Table 4:** Number of ORFs in each of the 14 multigene families identified in the fully sequenced avian poxvirus genomes.

Gene Family	ALPV2	ALPV	FWPV	MPPV2	MPPV	PEPV2	CNPV	SWPV2	MLPV	SWPV1	FP9	PEPV	FeP2	TKPV	FGPV
Ankyrin Repeat	33	48	31	78	62	49	51	46	47	50	22	33	26	16	45
B22R	6	6	6	9	7	6	6	7	7	6	5	5	4	1	4
N1R/p28	12	28	10	20	24	24	26	20	25	20	8	11	11	3	13
C4L/C10L	3	3	3	4	2	3	3	3	3	2	3	2	2	2	2
CC chemokine	4	5	4	7	4	5	5	5	5	6	4	1	4	2	6
C-type lectin	8	14	9	11	10	11	11	11	13	13	6	7	4	2	4
G protein-coupled receptor	3	4	3	4	4	4	4	4	4	4	2	3	2	2	3
HT motif	6	5	6	5	5	5	5	4	5	4	6	5	4	1	7
Ig-like domain	6	9	5	13	10	9	9	8	8	9	4	6	4	3	9
Serpin	6	5	5	5	5	5	5	5	5	5	5	4	4	3	5
EFc	3	2	3	3	2	2	2	2	1	2	2	1	1	1	1
TGF-β	1	5	1	5	4	5	5	4	6	3	1	1	1	1	1
β-NGF	2	2	2	2	2	2	2	2	2	2	2	0	0	2	3
IL-18 BP	2	3	1	3	3	3	3	3	3	3	1	1	0	2	0
TOTAL	95	139	89	169	144	133	137	124	134	129	71	80	67	41	103

## Data Availability

The complete genome sequence and associated datasets generated during this study were deposited in GenBank under the accession number OK348853.

## Supplementary Materials

The following supporting information can be downloaded at: https://www.mdpi.com/article/10.3390/v14020302/s1, Figure S1: Maximum likelihood (ML) phylogenetic tree from partial nucleotide sequences of the DNA polymerase gene of selected avipoxviruses; Figure S2: Maximum likelihood (ML) phylogenetic tree from partial nucleotide sequences of the P4b gene of selected avipoxviruses; Table S1: Albatrosspox virus 2 (ALPV2) genome annotations and comparative analysis of ORFs.
