# Supplementary material for: Evidence of a Possible Viral Host Switch Event in an Avipoxvirus Isolated from an Endangered Northern Royal Albatross (Diomedea sanfordi)
Source: Viruses. 2022 Feb 1;14(2):302. doi: 10.3390/v14020302 (PMC8880153; doi:10.3390/v14020302)
Supplement: Supplementary file 1 [file viruses-14-00302-s001.zip › viruses-1543909-supplementary.pdf]

viruses

Supplementary Materials

Evidence of a Possible Viral Host Switch Event in an Avipoxvirus Isolated from an Endangered Northern Royal Albatross (*Diomedea sanfordi*)

Subir Sarker [1,*], Timothy R. Bowden [2,3] and David B. Boyle [2]

[1] Department of Physiology, Anatomy and Microbiology, School of Life Sciences, La Trobe University, Melbourne, VIC 3086, Australia

[2] CSIRO Livestock Industries, Australian Animal Health Laboratory, Geelong, VIC 3220, Australia; timothy.bowden@csiro.au (T.R.B.); davidboyle48@gmail.com (D.B.B.)

[3] CSIRO Australian Animal Health Laboratory, Australian Centre for Disease Preparedness, Geelong, VIC 3220, Australia

* Correspondence: S.Sarker@latrobe.edu.au; Tel.: +61-3-9479-2317; Fax: +61-3-9479-1222

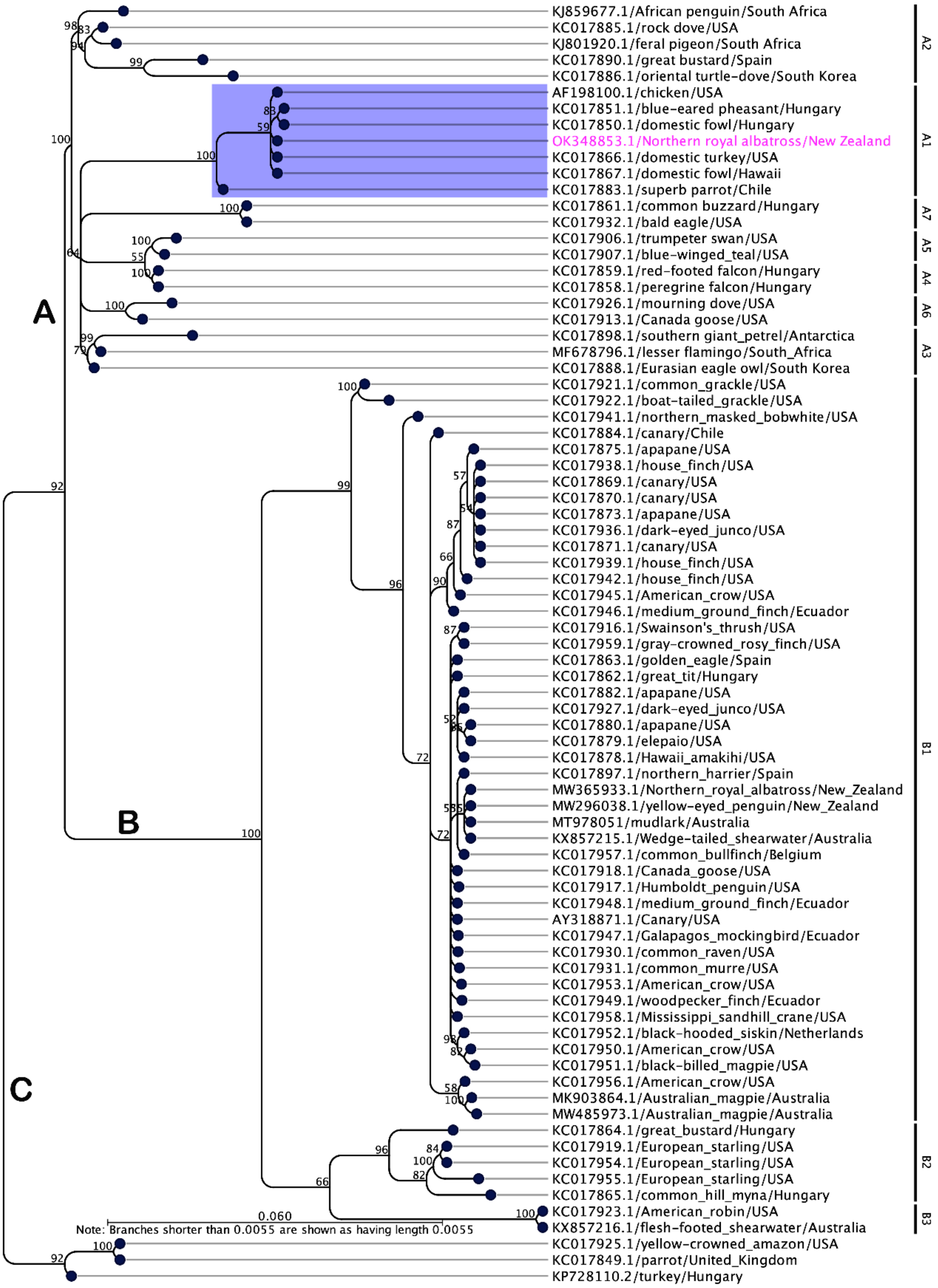

Figure S1: Maximum likelihood (ML) phylogenetic tree from partial nucleotide sequences of the DNA polymerase gene of selected avipoxviruses. Labels at branch tips refer to GenBank accession number/species/country of origin. The numbers on the left show bootstrap values as percentages. The relevant sub-clade A1 is highlighted using blue shading, whilst the position of ALPV22 is highlighted using pink text. The ML tree is displayed as a phylogram. The bootstrap value assigned to a node in the output tree is the percentage (0-100) of the bootstrap resamples which resulted in a tree containing the same subtree as that rooted at the node. Major clades and sub-clades are designated according to Gyuranecz *et al* (2013).

viruses

MDPI

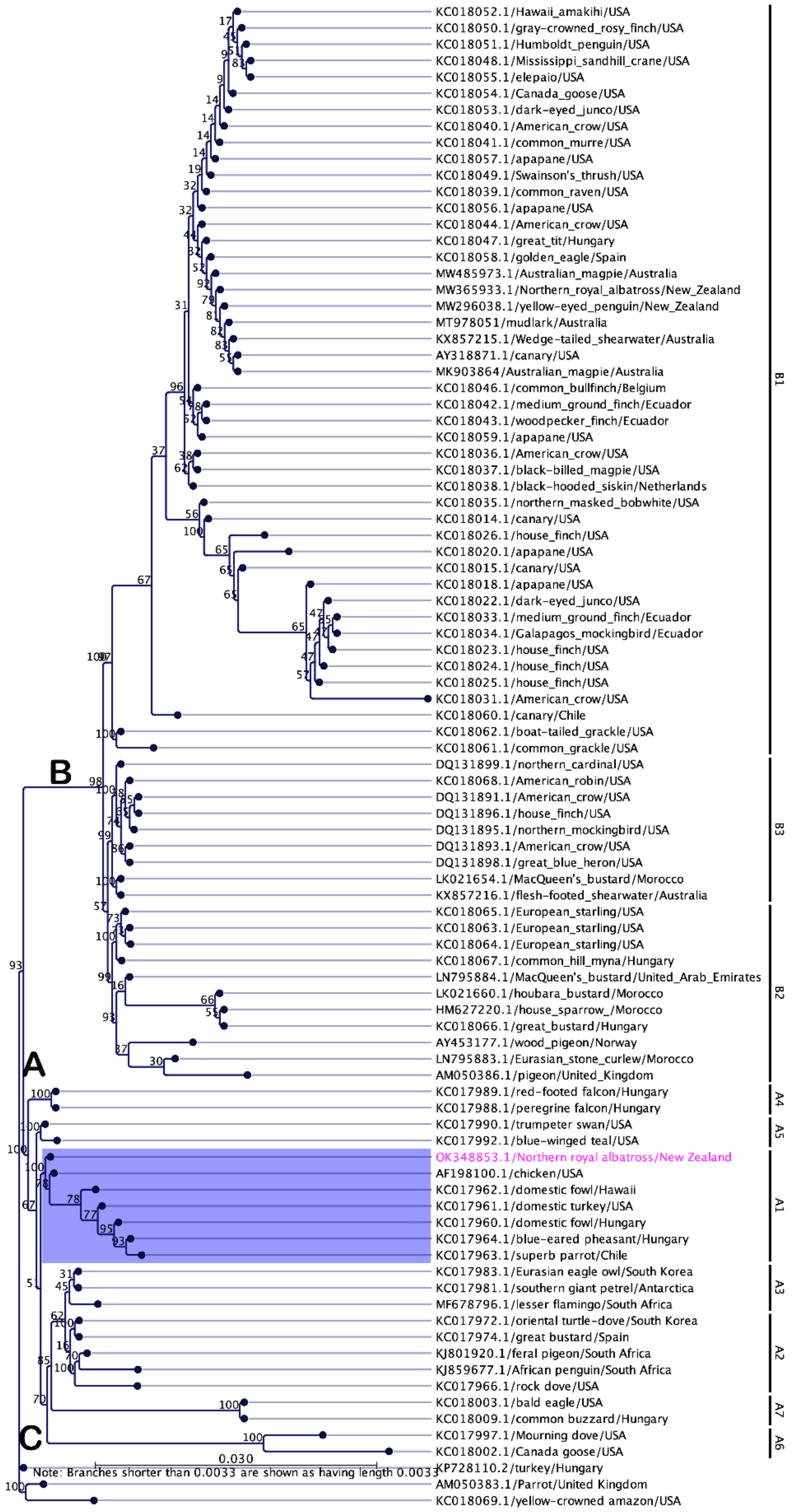

Figure S2: Maximum likelihood (ML) phylogenetic tree from partial nucleotide sequences of the P4b gene of selected avipoxviruses. Labels at branch tips refer to GenBank accession number/species/country of origin. The numbers on the left show bootstrap values as percentages. The relevant sub-clade A1 is highlighted with blue shading, whilst the position of ALPV22 is highlighted using pink text. The ML tree is displayed as a phylogram. The bootstrap value assigned to a node in the output tree is the percentage (0-100) of the bootstrap resamples which resulted in a tree containing the same subtree as that rooted at the node. Major clades and sub-clades are designated according to Gyuranecz *et al* (2013).

Table S1. Albatrosspox virus 2 (ALPV22) genome annotations and comparative analysis of ORFs.

ALPV2 Synteny	ALPV2 Genome Coordinates	FWPV Synteny	FWPV AA Size	ALPV2 AA Size	Best BLAST hits	ALPV2 AA Identity (%) Compared to Avipoxviruses	Notes
ALPV2-001	752-135	FWPV001	205	205	ORF FWPV001 hypothetical protein	100	
ALPV2-002	940-1068			42			hypothetical protein, unique to ALPV2
ALPV2-003	1179-1277			32			hypothetical protein, unique to ALPV2
ALPV2-004	1431-1529			32			hypothetical protein, unique to ALPV2
ALPV2-005	1628-2296	FWPV002	222	222	ORF FWPV002 hypothetical protein	100	
ALPV2-006	2314-2493			59	fpAU003 hypothetical protein	100	
ALPV2-007	2565-2705			46	MLPV323 hypothetical protein	56.8	
ALPV2-008	3132-2761	FWPV003	123	123	ORF FWPV003 C-type lectin gene family protein	100	
ALPV2-009	3386-3691	FWPV004	101	101	ORF FWPV004 hypothetical protein	100	
ALPV2-010	3850-3482	FWPV005	122	122	ORF FWPV005 EFc gene family protein	100	
ALPV2-011	4192-5448	FWPV006	418	418	ORF FWPV006 C4L/C10L-like gene family protein	100	
ALPV2-012	5429-5527			32			hypothetical protein, unique to ALPV2
ALPV2-013	5860-6078	FWPV007	72	72	ORF FWPV007 hypothetical protein	100	
ALPV2-014	5942-6445	FWPV008	167	167	ORF FWPV008 C-type lectin gene family protein	100	
ALPV2-015	6309-6509	FWPV009	66	66	ORF FWPV009 hypothetical protein	100	
ALPV2-016	7035-7181			48			hypothetical protein, unique to ALPV2, containing a transmembrane helix
ALPV2-017	8451-7384	FWPV010	355	355	ORF FWPV010 Serpin gene family protein	100	
ALPV2-018	9373-8537	FWPV011	278	278	ORF FWPV011 alpha-SNAP	100	

**ALPV2 Synteny**	**ALPV2 Genome Coordinates**	**FWPV Synteny**	**FWPV AA Size**	**ALPV2 AA Size**	**Best BLAST hits**	**ALPV2 AA Identity (%) Compared to Avipoxviruses**	**Notes**
ALPV2-019	9988-9845			47			hypothetical protein, unique to ALPV2, containing a transmembrane helix
ALPV2-020	9951-10076			41	fgpv_006 interleukin 10	52.8	
ALPV2-021	10221-10117			34			hypothetical protein, unique to ALPV2
ALPV2-022	10207-10302			31			hypothetical protein, unique to ALPV2
ALPV2-023	11294-10299	FWPV012	331	331	ORF FWPV012 Ankyrin repeat gene family protein	100	
ALPV2-024	11339-11446			35			hypothetical protein, unique to ALPV2, containing two transmembrane helices
ALPV2-025	11572-11456			38			hypothetical protein, unique to ALPV2
ALPV2-026	11699-11580			39	pepv_016 ankyrin repeat protein	70.3	
ALPV2-027	11909-12007			32			hypothetical protein, unique to ALPV2
ALPV2-028	12151-11969	FWPV013	60	60	ORF FWPV013 hypothetical protein	100	
ALPV2-029	13483-12170	FWPV014	437	437	ORF FWPV014 Ankyrin repeat gene family protein	100	
ALPV2-030	13482-13607			41			hypothetical protein, unique to ALPV2, containing a transmembrane helix
ALPV2-031	13737-13597			46			hypothetical protein, unique to ALPV2, containing two transmembrane helices
ALPV2-032	14437-13904	FWPV015	177	177	ORF FWPV015 hypothetical protein	100	
**ALPV2-033**	**15329-14613**	**FWPV016**	**238**	**238**	**ORF FWPV016 Ig-like domain**	**100**	
**ALPV2-034**	**16174-15437**	**FWPV017**	**245**	**245**	**ORF FWPV017 V-type Ig domain**	**100**	
ALPV2-035	18352-16250	FWPV018	700	700	ORF FWPV018 Ankyrin repeat gene family protein	100	

**ALPV2 Synteny**	**ALPV2 Genome Coordinates**	**FWPV Synteny**	**FWPV AA Size**	**ALPV2 AA Size**	**Best BLAST hits**	**ALPV2 AA Identity (%) Compared to Avipoxviruses**	**Notes**
ALPV2-036	18748-18434	FWPV019	104	104	ORF FWPV019 hypothetical protein	100	
ALPV2-037	18877-18755			40	gp024 hypothetical protein	91.3	
ALPV2-038	18886-18996			36			hypothetical protein, unique to ALPV2
ALPV2-039	19203-19048			51	fpAU022 hypothetical protein	100	
**ALPV2-040**	**20563-19283**	**FWPV020**	**426**	**426**	**ORF FWPV020 C4L/C10L-like gene family protein (vaccinia C10L)**	**100**	
**ALPV2-041**	**20654-21616**	**FWPV021**	**320**	**320**	**ORF FWPV021 G protein-coupled receptor gene family protein**	**100**	
**ALPV2-042**	**23372-21636**	**FWPV022**	**578**	**578**	**ORF FWPV022 Ankyrin repeat gene family protein**	**100**	
**ALPV2-043**	**24757-23453**	**FWPV023**	**434**	**434**	**ORF FWPV023 Ankyrin repeat gene family protein**	**100**	
**ALPV2-044**	**26613-24823**	**FWPV024**	**596**	**596**	**ORF FWPV024 Ankyrin repeat gene family protein**	**100**	
ALPV2-045	27327-26716	FWPV025	203	203	ORF FWPV025 hypothetical protein	100	
ALPV2-046	27623-27498			41			hypothetical protein, unique to ALPV2
ALPV2-047	29015-27705	FWPV026	436	436	ORF FWPV026 Ankyrin repeat gene family protein	100	
ALPV2-048	29057-30067	FWPV027	336	336	ORF FWPV027 G protein-coupled receptor gene family protein	100	
ALPV2-049	30118-30660	FWPV028	180	180	ORF FWPV028 hypothetical protein	100	
ALPV2-050	31137-31241			34			hypothetical protein, unique to ALPV2, containing a transmembrane helix
ALPV2-051	31657-31283	FWPV029	124	124	ORF FWPV029 Conserved hypothetical protein	100	
ALPV2-052	31670-31789			39			hypothetical protein, unique to ALPV2, containing a transmembrane helix
**ALPV2-053**	**34211-31758**	**FWPV030**	**817**	**817**	**ORF FWPV030 Alkaline phosphodiesterase-like protein**	**100**	

**ALPV2 Synteny**	**ALPV2 Genome Coordinates**	**FWPV Synteny**	**FWPV AA Size**	**ALPV2 AA Size**	**Best BLAST hits**	**ALPV2 AA Identity (%) Compared to Avipoxviruses**	**Notes**
**ALPV2-054**	**35361-34336**	**FWPV031**	**341**	**341**	**ORF FWPV031 Ankyrin repeat gene family protein**	**100**	
ALPV2-055	36119-35421	FWPV032	232	232	ORF FWPV032 DNase II	100	
ALPV2-056	36307-36498			63	FP-9 deoxyribonuclease II	100	
ALPV2-057	36867-36709			52	fpAU036 hypothetical protein	100	
ALPV2-058	37796-36933	FWPV033	287	287	ORF FWPV033 alpha-SNAP	100	
ALPV2-059	39084-37837	FWPV034	415	415	ORF FWPV034 Ankyrin repeat gene family protein	100	
**ALPV2-060**	**39675-39268**	**FWPV035**	**135**	**135**	**ORF FWPV035 hypothetical protein**	**100**	
ALPV2-061	40174-39713	FWPV036	153	153	ORF FWPV036 hypothetical protein	100	
**ALPV2-062**	**40667-40179**	**FWPV037**	**162**	**162**	**ORF FWPV037 hypothetical protein**	**100**	
ALPV2-063	41101-40664	FWPV038	145	145	ORF FWPV038 dUTP pyrophosphatase vaccinia F2L homolog	100	
**ALPV2-064**	**41679-41152**	**FWPV039**	**175**	**175**	**ORF FWPV039 Bcl-2 protein**	**100**	
**ALPV2-065**	**42737-41724**	**FWPV040**	**337**	**337**	**ORF FWPV040 Serpin gene family protein**	**100**	
ALPV2-066	42727-42849			40			hypothetical protein, unique to ALPV2, containing a transmembrane helix
ALPV2-067	43439-42819	FWPV041	206	206	ORF FWPV041 hypothetical protein	100	
ALPV2-068	43483-43740	FWPV042	85	85	ORF FWPV042 hypothetical protein	100	
**ALPV2-069**	**45218-43524**	**FWPV043**	**564**	**564**	**ORF FWPV043 DNA ligase**	**100**	
**ALPV2-070**	**46328-45252**	**FWPV044**	**358**	**358**	**ORF FWPV044 Serpin gene family protein**	**100**	
**ALPV2-071**	**47489-46377**	**FWPV046**	**370**	**370**	**ORF FWPV046 Hydroxysteroid dehydrogenase**	**100**	
ALPV2-072	47434-47655	FWPV045	73	73	ORF FWPV045 hypothetical protein	100	
**ALPV2-073**	**49379-47541**	**FWPV047**	**612**	**612**	**ORF FWPV047 Semaphorin vaccinia A39R homolog**	**100**	
ALPV2-074	49381-49533			50	fpAU052 hypothetical protein	100	

ALPV2 Synteny	ALPV2 Genome Coordinates	FWPV Synteny	FWPV AA Size	ALPV2 AA Size	Best BLAST hits	ALPV2 AA Identity (%) Compared to Avipoxviruses	Notes
ALPV2-075	49684-49523			53	fpAU053 hypothetical protein	100	
**ALPV2-076**	**49829-50614**	**FWPV048**	**261**	**261**	**ORF FWPV048 GNS1/SUR4 protein**	**100**	
ALPV2-077	***50690-51154***	***FWPV049***	***154***	***154***	***ORF FWPV049 Late transcription factor***	***100***	
ALPV2-078	***51175-52833***	***FWPV050***	***69***	***552***	***ORF FWPV050 Rifampicin resistance protein***	***100***	
ALPV2-079	***52865-53734***	***FWPV051***	***289***	***289***	***ORF FWPV051 mRNA capping enzyme, small subunit***	***100***	
ALPV2-080	***53809-55722***	***FWPV052***	***637***	***637***	***ORF FWPV052 NPH-I, transcription termination factor***	***100***	
ALPV2-081	***56403-55726***	***FWPV053***	***225***	***225***	***ORF FWPV053 mutT motif, gene expression regulator***	***100***	
**ALPV2-082**	**57082-56387**	**FWPV054**	**231**	**231**	**ORF FWPV054 mutT motif**	**100**	
ALPV2-083	58159-57332	FWPV055	275	275	ORF FWPV055 V-type Ig domain	100	
ALPV2-084	***58808-58323***	***FWPV056***	***161***	***161***	***ORF FWPV056 RNA polymerase subunit RPO18***	***100***	
ALPV2-085	***60696-58795***	***FWPV057***	***633***	***633***	***ORF FWPV057 Early transcription factor small subunit VETFS***	***100***	
ALPV2-086	63052-60677	FWPV058	791	791	ORF FWPV058 NTPase, DNA replication	100	
ALPV2-087	63786-63685			33			hypothetical protein, unique to ALPV2
ALPV2-088	63804-63896			30			hypothetical protein, unique to ALPV2, containing a transmembrane helix
ALPV2-089	63993-64652	FWPV059	219	219	ORF FWPV059 Deoxycytidine kinase	100	
ALPV2-090	65263-64697	FWPV060	188	188	ORF FWPV060 CC chemokine gene family protein	100	
ALPV2-091	65478-65609			43			hypothetical protein, unique to ALPV2
ALPV2-092	66026-65637	FWPV061	129	129	ORF FWPV061 CC chemokine gene family protein	100	
ALPV2-093	***66778-66122***	***FWPV062***	***218***	***218***	***ORF FWPV062 Uracil DNA glycosylase***	***100***	

ALPV2 Synteny	ALPV2 Genome Coordinates	FWPV Synteny	FWPV AA Size	ALPV2 AA Size	Best BLAST hits	ALPV2 AA Identity (%) Compared to Avipoxviruses	Notes
ALPV2-094	68028-66826	FWPV063	400	400	ORF FWPV063 hypothetical protein	100	
ALPV2-095	68401-68802	FWPV064	200	133	ORF FWPV064 Glutathione peroxidase	100	
**ALPV2-096**	**68806-69141**	**FWPV065**	**111**	**111**	**ORF FWPV065 hypothetical protein**	**100**	
ALPV2-097	69484-69116	FWPV066	122	122	ORF FWPV066 hypothetical protein	100	
ALPV2-098	69872-69600	FWPV067	90	90	ORF FWPV067 HT motif gene family protein	100	
ALPV2-099	69878-69970			30			hypothetical protein, unique to ALPV2
**ALPV2-100**	**70643-70242**	**FWPV068**	**133**	**133**	**ORF FWPV068 hypothetical protein**	**100**	
ALPV2-101	***71529-70717***	***FWPV069***	***270***	***270***	***ORF FWPV069 Virion protein***	***100***	
**ALPV2-102**	**71655-72476**	**FWPV070**	**273**	**273**	**ORF FWPV070 T10 gene product**	**100**	
ALPV2-103	72622-72491			43	pepv_073 hypothetical protein	86.1	
**ALPV2-104**	**73750-72881**	**FWPV071**	**289**	**289**	**ORF FWPV071 Conserved hypothetical protein**	**100**	
ALPV2-105	74497-73937	FWPV072	186	186	ORF FWPV072 beta-Nerve growth factor	100	
ALPV2-106	75043-74519	FWPV073	174	174	ORF FWPV073 Interleukin binding protein	100	
ALPV2-107	75094-75228			44			hypothetical protein, unique to ALPV2, containing two transmembrane helices
ALPV2-108	75612-75298	FWPV074	104	104	ORF FWPV074 hypothetical protein	100	
**ALPV2-109**	**76215-75616**	**FWPV075**	**199**	**199**	**ORF FWPV075 N1R/p28 gene family protein**	**100**	
ALPV2-110	76718-76284	FWPV076	144	144	ORF FWPV076 beta-Nerve growth factor	100	
ALPV2-111	***76830-77207***	***FWPV077***	***125***	***125***	***ORF FWPV077 Glutaredoxin vaccinia G4L and Molluscum contagiosum virus MC059L homolog***	***100***	
ALPV2-112	***77857-77180***	***FWPV079***	***225***	***225***	***ORF FWPV079 Putative elongation factor***	***100***	
ALPV2-113	***77851-78162***	***FWPV078***	***103***	***103***	***ORF FWPV078 conserved hypothetical protein***	***100***	
ALPV2-114	79280-78189	FWPV080	363	363	ORF FWPV080 Transforming growth factor-beta	100	
ALPV2-115	***79352-81232***	***FWPV081***	***626***	***626***	***ORF FWPV081 Metalloprotease***	***100***	

ALPV2 Synteny	ALPV2 Genome Coordinates	FWPV Synteny	FWPV AA Size	ALPV2 AA Size	Best BLAST hits	ALPV2 AA Identity (%) Compared to Avipoxviruses	Notes
ALPV2-116	***83264-81216***	***FWPV082***	***682***	***682***	***ORF FWPV082 RNA helicase/NPH-II***	***100***	
ALPV2-117	***83297-84562***	***FWPV083***	***421***	***421***	***ORF FWPV083 Virion core protein***	***100***	
ALPV2-118	***84565-85737***	***FWPV084***	***390***	***390***	***ORF FWPV084 DNA-binding protein***	***100***	
ALPV2-119	***85738-85983***	***FWPV085***	***81***	***81***	***ORF FWPV085 putative IMV membrane protein***	***100***	
**ALPV2-120**	**85993-86544**	**FWPV086**	**183**	**183**	**ORF FWPV086 Thymidine kinase**	**100**	
ALPV2-121	***86621-86896***	***FWPV087***	***91***	***91***	***ORF FWPV087 HT motif gene family protein***	***100***	
ALPV2-122	***86929-87801***	***FWPV088***	***290***	***290***	***ORF FWPV088 DNA-binding phosphoprotein***	***100***	
ALPV2-123	***87802-87999***	***FWPV089***	***65***	***65***	***ORF FWPV089 conserved hypothetical protein***	***100***	
ALPV2-124	***88006-88941***	***FWPV090***	***311***	***311***	***ORF FWPV090 DNA-binding virion protein***	***100***	
ALPV2-125	88942-89046			34	fpO3L, orthologue of vaccinia O3L & MC043.1L	100	
**ALPV2-126**	**89106-91076**	**FWPV091**	**656**	**656**	**ORF FWPV091 conserved hypothetical protein**	**100**	
**ALPV2-127**	**91018-91413**	**FWPV092**	**131**	**131**	**ORF FWPV092 virion core protein**	**100**	
ALPV2-128	***91694-91410***	***FWPV093***	***94***	***94***	***ORF FWPV093 putative IMV redox protein, virus assembly***	***100***	
ALPV2-129	***91721-94687***	***FWPV094***	***988***	***988***	***ORF FWPV094 DNA polymerase***	***100***	
ALPV2-130	***95497-94679***	***FWPV095***	***272***	***272***	***ORF FWPV095 putative membrane protein***	***100***	
ALPV2-131	***97205-95490***	***FWPV096***	***571***	***571***	***ORF FWPV096 conserved hypothetical protein***	***100***	
ALPV2-132	97249-97347			32			hypothetical protein, unique to ALPV2, containing a transmembrane helix
ALPV2-133	103069-97331	FWPV097	1912	1912	ORF FWPV097 variola B22R gene family protein	100	
ALPV2-134	103075-103167			30			hypothetical protein, unique to ALPV2
ALPV2-135	108543-103135	FWPV098	1802	1802	ORF FWPV098 variola B22R gene family protein	100	
ALPV2-136	108533-108625			30			hypothetical protein, unique to ALPV2
ALPV2-137	114540-108784	FWPV099	1918	1918	ORF FWPV099 variola B22R gene family protein	98.4	

ALPV2 Synteny	ALPV2 Genome Coordinates	FWPV Synteny	FWPV AA Size	ALPV2 AA Size	Best BLAST hits	ALPV2 AA Identity (%) Compared to Avipoxviruses	Notes
ALPV2-138	***114607-115155***	***FWPV100***	***182***	***182***	***ORF FWPV100 RNA polymerase subunit, RPO30***	***100***	
ALPV2-139	***115208-117361***	***FWPV101***	***717***	***717***	***ORF FWPV101 conserved hypothetical protein***	***100***	
ALPV2-140	***117348-118766***	***FWPV102***	***472***	***472***	***ORF FWPV102 Poly(A) polymerase large subunit, PAPL***	***100***	
ALPV2-141	***119104-118760***	***FWPV103***	***114***	***114***	***ORF FWPV103 DNA-binding, virion core phosphoprotein***	***100***	
**ALPV2-142**	**119181-119813**	**FWPV104**	**210**	**210**	**ORF FWPV104 conserved hypothetical protein**	**100**	
**ALPV2-143**	**119938-120384**	**FWPV105**	**148**	**148**	**ORF FWPV105 conserved hypothetical protein**	**100**	
ALPV2-144	120435-120587			50	fpAU113 hypothetical protein	100	
ALPV2-145	120674-120889	FWPV106	71	71	ORF FWPV106 hypothetical protein	100	
ALPV2-146	126270-120937	FWPV107	1777	1777	ORF FWPV107 variola B22R gene family protein	100	
ALPV2-147	***126452-127585***	***FWPV108***	***377***	***377***	***ORF FWPV108 putative palmitylated EEV envelope lipase***	***100***	
ALPV2-148	***127623-129515***	***FWPV109***	***630***	***630***	***ORF FWPV109 putative EEV maturation protein***	***100***	
**ALPV2-149**	**129555-130910**	**FWPV110**	**451**	**451**	**ORF FWPV110 conserved hypothetical protein**	**100**	
ALPV2-150	***130988-132322***	***FWPV111***	***444***	***444***	***ORF FWPV111 Serine/threonine protein kinase, virus assembly***	***100***	
ALPV2-151	***132297-132938***	***FWPV112***	***213***	***213***	***ORF FWPV112 Putative membrane protein***	***100***	
**ALPV2-152**	**133029-133229**	**FWPV113**	**66**	**66**	**ORF FWPV113 conserved hypothetical protein**	**100**	
ALPV2-153	133441-133343			32			hypothetical protein, unique to ALPV2
ALPV2-154	133409-133567			52	fpAU122 hypothetical protein	100	
ALPV2-155	133598-134149	FWPV114	183	183	ORF FWPV114 HAL3 domain	100	
ALPV2-156	134308-134174			44			hypothetical protein, unique to ALPV2, containing a transmembrane helix
ALPV2-157	135035-134904			43			hypothetical protein, unique to ALPV2, containing a transmembrane helix

ALPV2 Synteny	ALPV2 Genome Coordinates	FWPV Synteny	FWPV AA Size	ALPV2 AA Size	Best BLAST hits	ALPV2 AA Identity (%) Compared to Avipoxviruses	Notes
ALPV2-158	135316-136944	FWPV115	542	542	ORF FWPV115 Ankyrin repeat gene family protein	100	
ALPV2-159	136966-137328	FWPV116	120	120	ORF FWPV116 CC chemokine gene family protein	100	
ALPV2-160	137376-137540			54	fpAU126 hypothetical protein	100	
ALPV2-161	137625-137735			36			hypothetical protein, unique to ALPV2
ALPV2-162	137780-137685			31			hypothetical protein, unique to ALPV2, containing a transmembrane helix
ALPV2-163	***137874-139196***	***FWPV117***	***440***	***440***	***ORF FWPV117 conserved hypothetical protein***	***100***	
ALPV2-164	***139198-139389***	***FWPV118***	***63***	***63***	***ORF FWPV118 RNA polymerase subunit, RP07***	***100***	
ALPV2-165	***139389-139955***	***FWPV119***	***188***	***188***	***ORF FWPV119 conserved hypothetical protein***	***100***	
ALPV2-166	***140951-139920***	***FWPV120***	***343***	***343***	***ORF FWPV120 Virion core protein***	***100***	
ALPV2-167	141355-141720	FWPV121	121	121	ORF FWPV121 CC chemokine gene family protein	100	
ALPV2-168	142159-142043			38			hypothetical protein, unique to ALPV2
ALPV2-169	142163-142261			32			hypothetical protein, unique to ALPV2
ALPV2-170	148234-142622	FWPV122	1870	1870	ORF FWPV122 variola B22R gene family protein	100	
ALPV2-171	153565-148265	FWPV123	1766	1766	ORF FWPV123 variola B22R gene family protein	100	
ALPV2-172	154043-154168			41	CNPV219 N1R/p28-like protein	68.3	
ALPV2-173	154534-154785	FWPV124	289	83	ORF FWPV124 N1R/p28 gene family protein	96.3	
ALPV2-174	154858-154715	FWPV124	289	47	ORF FWPV124 N1R/p28 gene family protein	100	
ALPV2-175	156309-157346	FWPV125	345	345	ORF FWPV125 V-type Ig domain	100	
ALPV2-176	157582-157487			31			hypothetical protein, unique to ALPV2

ALPV2 Synteny	ALPV2 Genome Coordinates	FWPV Synteny	FWPV AA Size	ALPV2 AA Size	Best BLAST hits	ALPV2 AA Identity (%) Compared to Avipoxviruses	Notes
ALPV2-177	157738-157643			31			hypothetical protein, unique to ALPV2
ALPV2-178	***157768-158550***	***FWPV126***	***260***	***260***	***ORF FWPV126 Late transcription factor VLTF-1***	***100***	
ALPV2-179	***158563-159573***	***FWPV127***	***336***	***336***	***ORF FWPV127 Myristylated protein***	***100***	
ALPV2-180	***159574-160305***	***FWPV128***	***243***	***243***	***ORF FWPV128 Myristylated membrane protein***	***100***	
ALPV2-181	***160340-160630***	***FWPV129***	***96***	***96***	***ORF FWPV129 conserved hypothetical protein***	***100***	
ALPV2-182	***161525-160620***	***FWPV130***	***301***	***301***	***ORF FWPV130 conserved hypothetical protein***	***100***	
ALPV2-183	***161551-162312***	***FWPV131***	***253***	***253***	***ORF FWPV131 DNA-binding virion core protein VP8***	***100***	
ALPV2-184	***162313-162702***	***FWPV132***	***129***	***129***	***ORF FWPV132 Putative membrane protein***	***100***	
ALPV2-185	***162653-163099***	***FWPV133***	***148***	***148***	***ORF FWPV133 185 putative IMV membrane protein***	***100***	
ALPV2-186	***163132-164058***	***FWPV134***	***308***	***308***	***ORF FWPV134 Poly(A) polymerase small subunit PAPS***	***100***	
ALPV2-187	***164055-164615***	***FWPV135***	***186***	***186***	***ORF FWPV135 RNA polymerase subunit RP022***	***100***	
ALPV2-188	***165018-164605***	***FWPV136***	***137***	***137***	***ORF FWPV136 conserved hypothetical protein***	***100***	
ALPV2-189	***165059-168922***	***FWPV137***	***1287***	***1287***	***ORF FWPV137 RNA polymerase subunit, RPO147***	***100***	
ALPV2-190	***169431-168931***	***FWPV138***	***166***	***166***	***ORF FWPV138 Protein-tyrosine phosphatase***	***100***	
ALPV2-191	***169447-170019***	***FWPV139***	***190***	***190***	***ORF FWPV139 conserved hypothetical protein***	***100***	
ALPV2-192	***171183-170200***	***FWPV140***	***327***	***327***	***ORF FWPV140 Virion envelope protein, p35***	***100***	
ALPV2-193	***173580-171184***	***FWPV141***	***798***	***798***	***ORF FWPV141 RNA polymerase associated protein RAP94***	***100***	
ALPV2-194	***173724-174248***	***FWPV142***	***174***	***174***	***ORF FWPV142 Late transcription factor VLTF-4***	***100***	
ALPV2-195	***174249-175199***	***FWPV143***	***316***	***316***	***ORF FWPV143 DNA topoisomerase***	***100***	
ALPV2-196	***175204-175662***	***FWPV144***	***152***	***152***	***ORF FWPV144 conserved hypothetical protein***	***100***	
**ALPV2-197**	**175936-175625**	**FWPV145**	**103**	**103**	**ORF FWPV145 conserved hypothetical protein**	**100**	

ALPV2 Synteny	ALPV2 Genome Coordinates	FWPV Synteny	FWPV AA Size	ALPV2 AA Size	Best BLAST hits	ALPV2 AA Identity (%) Compared to Avipoxviruses	Notes
ALPV2-198	***175944-178499***	***FWPV146***	***851***	***851***	***ORF FWPV146 mRNA Capping enzyme, large subunit***	***100***	
ALPV2-199	178558-178872	FWPV147	104	104	ORF FWPV147 HT motif gene family protein	100	
ALPV2-200	***179288-178869***	***FWPV148***	***139***	***139***	***ORF FWPV148 Virion protein***	***100***	
ALPV2-201	179556-180116	FWPV149	186	186	ORF FWPV149 hypothetical protein	100	
ALPV2-202	180183-181013	FWPV150	276	276	ORF FWPV150 N1R/p28 gene family protein	100	
**ALPV2-203**	**181053-181760**	**FWPV151**	**235**	**235**	**ORF FWPV151 Deoxycytidine kinase**	**100**	
ALPV2-204	182153-181770	FWPV152	127	127	ORF FWPV152 HT motif gene family protein	100	
ALPV2-205	182252-182878	FWPV153	208	208	ORF FWPV153 hypothetical protein	100	
ALPV2-206	183103-183555	FWPV154	150	150	ORF FWPV154 hypothetical protein	100	
ALPV2-207	183584-184810	FWPV155	408	408	ORF FWPV155 N1R/p28 gene family protein	100	
ALPV2-208	184859-185020			53	fpAU166 hypothetical protein	100	
ALPV2-209	185066-185464	FWPV156	132	132	ORF FWPV156 HT motif gene family protein	100	
ALPV2-210	185510-186445	FWPV157	311	311	ORF FWPV157 N1R/p28 gene family protein	100	
ALPV2-211	186535-187929	FWPV158	464	464	ORF FWPV158 Photolyase	100	
ALPV2-212	188068-188793	FWPV159	241	241	ORF FWPV159 N1R/p28 gene family protein	100	
ALPV2-213	188922-188800			40			hypothetical protein, unique to ALPV2, containing a transmembrane helix
ALPV2-214	188874-189344	FWPV160	156	156	ORF FWPV160 hypothetical protein	100	
ALPV2-215	189392-189865	FWPV161	157	157	ORF FWPV161 N1R/p28 gene family protein	100	
ALPV2-216	190305-190460			51	fpAU173 hypothetical protein	100	
ALPV2-217	190556-190714			52	fpAU174 N1R/p28 family protein	100	
ALPV2-218	190845-190970			41			hypothetical protein, unique to ALPV2

ALPV2 Synteny	ALPV2 Genome Coordinates	FWPV Synteny	FWPV AA Size	ALPV2 AA Size	Best BLAST hits	ALPV2 AA Identity (%) Compared to Avipoxviruses	Notes
ALPV2-219	191190-191077			37			hypothetical protein, unique to ALPV2, containing a transmembrane helix
ALPV2-220	191243-193054	FWPV162	603	603	ORF FWPV162 Ankyrin repeat gene family protein	100	
ALPV2-221	193108-193010			32			hypothetical protein, unique to ALPV2
ALPV2-222	193288-193118			56	fpAU176 hypothetical protein	100	
ALPV2-223	193287-194078	FWPV163	263	263	ORF FWPV163 N1R/p28 gene family protein	100	
ALPV2-224	194369-194265			34			hypothetical protein, unique to ALPV2, containing a transmembrane helix
ALPV2-225	194571-194437			44			hypothetical protein, unique to ALPV2, containing a transmembrane helix
ALPV2-226	194570-195685	FWPV164	371	371	ORF FWPV164 hypothetical protein	96.9	
ALPV2-227	196036-195935			33			hypothetical protein, unique to ALPV2
ALPV2-228	***197429-196752***	***FWPV165***	***225***	***225***	***ORF FWPV165 Late transcription factor, VLTF-3***	***100***	
ALPV2-229	***197644-197426***	***FWPV166***	***72***	***72***	***ORF FWPV166 virion redox protein***	***100***	
ALPV2-230	***199632-197659***	***FWPV167***	***657***	***657***	***ORF FWPV167 Virion core protein P4b***	***100***	
ALPV2-231	***199799-199695***		***288***	***34***	***ORF FWPV168 Immunodominant virion protein***	***100***	
ALPV2-232	***200499-200155***	***FWPV168***	***288***	***114***	***ORF FWPV168 Immunodominant virion protein***	***100***	
ALPV2-233	***200538-201041***	***FWPV169***	***167***	***167***	***ORF FWPV169 RNA polymerase subunit RPO19***	***100***	
ALPV2-234	***202160-201036***	***FWPV170***	***374***	***374***	***ORF FWPV170 conserved hypothetical protein***	***100***	
ALPV2-235	***204293-202164***	***FWPV171***	***709***	***709***	***ORF FWPV171 Early transcription factor large subunit, VETFL***	***99.9***	
ALPV2-236	***204359-205264***	***FWPV172***	***301***	***301***	***ORF FWPV172 Intermediate transcription factor, VITF-3***	***100***	

ALPV2 Synteny	ALPV2 Genome Coordinates	FWPV Synteny	FWPV AA Size	ALPV2 AA Size	Best BLAST hits	ALPV2 AA Identity (%) Compared to Avipoxviruses	Notes
ALPV2-237	***205456-205226***	***FWPV173***	***76***	***76***	***ORF FWPV173 putative IMV membrane protein***	***100***	
ALPV2-238	***208132-205457***	***FWPV174***	***891***	***891***	***ORF FWPV174 Virion core protein P4a***	***100***	
ALPV2-239	***208150-208974***	***FWPV175***	***274***	***274***	***ORF FWPV175 conserved hypothetical protein***	***100***	
ALPV2-240	***209490-208975***	***FWPV176***	***171***	***171***	***ORF FWPV176 Virion protein***	***100***	
ALPV2-241	209505-209711	FWPV177	68	68	ORF FWPV177 conserved hypothetical protein	100	
ALPV2-242	***209916-209701***	***FWPV178***	***71***	***71***	***ORF FWPV178 Virion protein***	***100***	
ALPV2-243	***210258-209983***	***FWPV179***	***91***	***91***	***ORF FWPV179 putative IMV membrane protein***	***100***	
ALPV2-244	210436-210275			53	fpAU195 A14.5L putative IMV membrane virulence protein	100	
ALPV2-245	***210744-210451***	***FWPV180***	***97***	***97***	***ORF FWPV180 conserved hypothetical protein***	***100***	
ALPV2-246	***211837-210728***	***FWPV181***	***369***	***369***	***ORF FWPV181 Putative myristylated membrane protein***	***100***	
ALPV2-247	***212449-211853***	***FWPV182***	***198***	***198***	***ORF FWPV182 Phosphorylated virion membrane protein***	***100***	
ALPV2-248	***212464-213852***	***FWPV183***	***462***	***462***	***ORF FWPV183 DNA helicase, transcriptional elongation***	***100***	
ALPV2-249	***214086-213820***	***FWPV184***	***88***	***88***	***ORF FWPV184 conserved hypothetical protein***	***100***	
ALPV2-250	***214435-214094***	***FWPV186***	***113***	***113***	***ORF FWPV186 conserved hypothetical protein***	***100***	
ALPV2-251	***214434-215735***	***FWPV185***	***433***	***433***	***ORF FWPV185 DNA polymerase processivity factor***	***100***	
ALPV2-252	***215735-216205***	***FWPV187***	***156***	***156***	***ORF FWPV187 Holliday junction resolvase protein***	***100***	
ALPV2-253	***216216-217367***	***FWPV188***	***383***	***383***	***ORF FWPV188 Intermediate transcription factor, VITF-3***	***100***	
ALPV2-254	***217394-220879***	***FWPV189***	***1161***	***1161***	***ORF FWPV189 RNA polymerase subunit, RPO132***	***100***	
**ALPV2-255**	**222678-220816**	**FWPV190**	**620**	**620**	**ORF FWPV190 A type inclusion-like protein**	**100**	
**ALPV2-256**	**224138-222714**	**FWPV191**	**474**	**474**	**ORF FWPV191 A type inclusion protein**	**100**	
ALPV2-257	***224564-224139***	***FWPV192***	***141***	***141***	***ORF FWPV192 conserved hypothetical protein***	***100***	

ALPV2 Synteny	ALPV2 Genome Coordinates	FWPV Synteny	FWPV AA Size	ALPV2 AA Size	Best BLAST hits	ALPV2 AA Identity (%) Compared to Avipoxviruses	Notes
ALPV2-258	***225487-224579***	***FWPV193***	***302***	***302***	***ORF FWPV193 RNA polymerase subunit, RP035***	***100***	
ALPV2-259	***225686-225462***	***FWPV194***	***74***	***74***	***ORF FWPV194 conserved hypothetical protein***	***100***	
ALPV2-260	225855-225739			38	fp9.194.1 A30.5L orthologue	100	
ALPV2-261	225865-226206	FWPV195	113	113	ORF FWPV195 conserved hypothetical protein	100	
**ALPV2-262**	**226207-226569**	**FWPV196**	**120**	**120**	**ORF FWPV196 conserved hypothetical protein**	**100**	
ALPV2-263	***227463-226558***	***FWPV197***	***301***	***301***	***ORF FWPV197 Virion assembly protein***	***100***	
ALPV2-264	***227652-228173***	***FWPV198***	***173***	***173***	***ORF FWPV198 C-type lectin-like protein***	***100***	
ALPV2-265	228228-228887	FWPV199	219	219	ORF FWPV199 V-type Ig domain	100	
ALPV2-266	228862-229659	FWPV200	265	265	ORF FWPV200 V-type Ig domain	100	
**ALPV2-267**	**229680-230531**	**FWPV201**	**283**	**283**	**ORF FWPV201 conserved hypothetical protein**	**100**	
ALPV2-268	230717-230415	FWPV202	100	100	ORF FWPV202 hypothetical protein	100	
**ALPV2-269**	**230854-231711**	**FWPV203**	**285**	**285**	**ORF FWPV203 Tyrosine protein kinase**	**100**	
ALPV2-270	231747-232775	FWPV204	342	342	ORF FWPV204 Serpin gene family protein	100	
**ALPV2-271**	**233434-232778**	**FWPV205**	**218**	**218**	**ORF FWPV205 conserved hypothetical protein**	**100**	
ALPV2-272	233549-234475	FWPV206	308	308	ORF FWPV206 G protein-coupled receptor gene family protein	100	
**ALPV2-273**	**234485-234787**	**FWPV207**	**100**	**100**	**ORF FWPV207 conserved hypothetical protein**	**100**	
ALPV2-274	234925-235080			51	fpAU223 hypothetical protein	100	
ALPV2-275	235177-235019			52	fpAU224 hypothetical protein	100	
ALPV2-276	235414-235506			30			hypothetical protein, unique to ALPV2
**ALPV2-277**	**235673-235876**	**FWPV208**	**67**	**67**	**ORF FWPV208 conserved hypothetical protein**	**100**	
ALPV2-278	236534-236142	FWPV209	130	130	ORF FWPV209 HT motif gene family protein	100	
ALPV2-279	237038-237238	FWPV210	66	66	ORF FWPV210 hypothetical protein	100	
**ALPV2-280**	**237177-237554**	**FWPV211**	**125**	**125**	**ORF FWPV211 EGF-like protein vaccinia C11R**	**100**	

ALPV2 Synteny	ALPV2 Genome Coordinates	FWPV Synteny	FWPV AA Size	ALPV2 AA Size	Best BLAST hits	ALPV2 AA Identity (%) Compared to Avipoxviruses	Notes
**ALPV2-281**	**237557-238468**	**FWPV212**	**303**	**303**	**ORF FWPV212 serine/threonine protein kinase**	**100**	
**ALPV2-282**	**238519-239007**	**FWPV213**	**162**	**162**	**ORF FWPV213 conserved hypothetical protein**	**100**	
ALPV2-283	239102-239004			32			hypothetical protein, unique to ALPV2
**ALPV2-284**	**239346-239720**	**FWPV214**	**124**	**124**	**ORF FWPV214 putative interleukin binding protein**	**100**	
ALPV2-285	239824-239726			32			hypothetical protein, unique to ALPV2
ALPV2-286	239833-240057	FWPV215	74	74	ORF FWPV215 conserved hypothetical protein	100	
ALPV2-287	240201-240356			51	fpAU233 hypothetical protein	100	
ALPV2-288	240492-240370			40			hypothetical protein, unique to ALPV2, containing a transmembrane helix
ALPV2-289	240452-241342	FWPV216	296	296	ORF FWPV216 Ankyrin repeat gene family protein	100	
ALPV2-290	241621-241725			34			hypothetical protein, unique to ALPV2
ALPV2-291	241771-242757	FWPV217	328	328	ORF FWPV217 hypothetical protein	100	
ALPV2-292	242797-244182	FWPV218	461	461	ORF FWPV218 Ankyrin repeat gene family protein	100	
**ALPV2-293**	**244212-245516**	**FWPV219**	**434**	**434**	**ORF FWPV219 Ankyrin repeat gene family protein**	**100**	
ALPV2-294	245521-245168	FWPV220	117	117	ORF FWPV220 hypothetical protein	100	
ALPV2-295	246069-245518	FWPV221	183	183	ORF FWPV221 vaccinia A47L homolog	100	
ALPV2-296	246157-248400	FWPV222	747	747	ORF FWPV222 Ankyrin repeat gene family protein	100	
ALPV2-297	248612-249037	FWPV223	141	141	ORF FWPV223 Ankyrin repeat gene family protein	100	
ALPV2-298	249042-249482	FWPV224	146	146	ORF FWPV224 Ankyrin repeat gene family protein	100	
ALPV2-299	249662-249537			41	CNPV298 ankyrin repeat protein	56.1	

**ALPV2 Synteny**	**ALPV2 Genome Coordinates**	**FWPV Synteny**	**FWPV AA Size**	**ALPV2 AA Size**	**Best BLAST hits**	**ALPV2 AA Identity (%) Compared to Avipoxviruses**	**Notes**
ALPV2-300	249859-250173	FWPV225	104	104	ORF FWPV225 vaccinia B20R homolog	100	
ALPV2-301	250176-251054	FWPV226	292	292	ORF FWPV226 serine/threonine protein kinase	100	
ALPV2-302	251107-252192	FWPV227	361	361	ORF FWPV227 Ankyrin repeat gene family protein	100	
ALPV2-303	252282-253859	FWPV228	525	525	ORF FWPV228 Ankyrin repeat gene family protein	100	
ALPV2-304	254106-254011			31			hypothetical protein, unique to ALPV2
ALPV2-305	254521-254613			30			hypothetical protein, unique to ALPV2
ALPV2-306	254839-254931			30			hypothetical protein, unique to ALPV2
ALPV2-307	255125-255033			30	FWPV229 hypothetical protein	100	
ALPV2-308	255576-255151	FWPV229	141	141	ORF FWPV229 hypothetical protein	77.8	
ALPV2-309	255708-256274	FWPV230	188	188	ORF FWPV230 Ankyrin repeat gene family protein	100	
ALPV2-310	256238-257008	FWPV231	256	256	ORF FWPV231 Ankyrin repeat gene family protein	100	
ALPV2-311	257260-257159			33			hypothetical protein, unique to ALPV2, containing a transmembrane helix
**ALPV2-312**	**257275-258723**	**FWPV232**	**482**	**482**	**ORF FWPV232 Ankyrin repeat gene family protein**	**100**	
ALPV2-313	258766-260304	FWPV233	512	512	ORF FWPV233 Ankyrin repeat gene family protein	100	
ALPV2-314	260335-261621	FWPV234	428	428	ORF FWPV234 Ankyrin repeat gene family protein	100	
ALPV2-315	261640-262071	FWPV235	143	143	ORF FWPV235 C-type lectin gene family protein	100	
ALPV2-316	262074-262916	FWPV236	280	280	ORF FWPV236 N1R/p28 gene family protein	100	
ALPV2-317	263128-262925	FWPV237	67	67	ORF FWPV237 hypothetical protein	98.5	
ALPV2-318	263228-263088			46	fgpv_256 putative P-type ATPase	46.8	

ALPV2 Synteny	ALPV2 Genome Coordinates	FWPV Synteny	FWPV AA Size	ALPV2 AA Size	Best BLAST hits	ALPV2 AA Identity (%) Compared to Avipoxviruses	Notes
ALPV2-319	263735-263244	FWPV239	163	163	ORF FWPV239 C-type lectin gene family protein	100	
ALPV2-320	263681-263866	FWPV238	61	61	ORF FWPV238 hypothetical protein	100	
ALPV2-321	263854-265086	FWPV240	410	410	ORF FWPV240 Ankyrin repeat gene family protein	100	
ALPV2-322	265485-266045	FWPV241	186	186	ORF FWPV241 Ankyrin repeat gene family protein	55.9	
ALPV2-323	266251-266138			37			hypothetical protein, unique to ALPV2, containing a transmembrane helix
ALPV2-324	266414-266527			37			hypothetical protein, unique to ALPV2
ALPV2-325	266568-266473			31			hypothetical protein, unique to ALPV2
ALPV2-326	266631-266726			31			hypothetical protein, unique to ALPV2
ALPV2-327	267063-266902			53	fpAU260 hypothetical protein	100	
ALPV2-328	267397-268473	FWPV242	358	358	ORF FWPV242 Ankyrin repeat gene family protein	100	
ALPV2-329	268542-269330	FWPV243	262	262	ORF FWPV243 Ankyrin repeat gene family protein	100	
ALPV2-330	269624-269743			39			hypothetical protein, unique to ALPV2
ALPV2-331	269997-269815			60	fpAU263 hypothetical protein	100	
ALPV2-332	270204-270037			55	fpAU264 hypothetical protein	100	
ALPV2-333	270303-272309	FWPV244	668	668	ORF FWPV244 Ankyrin repeat gene family protein	99.9	
ALPV2-334	273628-272318	FWPV245	436	436	ORF FWPV245 Ankyrin repeat gene family protein	100	
ALPV2-335	274010-275788	FWPV246	592	592	ORF FWPV246 Ankyrin repeat gene family protein	100	
ALPV2-336	275869-275777			30			hypothetical protein, unique to ALPV2

ALPV2 Synteny	ALPV2 Genome Coordinates	FWPV Synteny	FWPV AA Size	ALPV2 AA Size	Best BLAST hits	ALPV2 AA Identity (%) Compared to Avipoxviruses	Notes
ALPV2-337	275841-276215	FWPV247	124	124	ORF FWPV247 EFc gene family protein	100	
ALPV2-338	276327-276782	FWPV248	151	151	ORF FWPV248 N1R/p28 gene family protein	100	
ALPV2-339	277163-277480	FWPV249	105	105	ORF FWPV249 hypothetical protein	100	
ALPV2-340	277664-277548			38			hypothetical protein, unique to ALPV2, containing a transmembrane helix
ALPV2-341	278131-277709	FWPV250	140	140	ORF FWPV250	100	
ALPV2-342	278247-278384	FWPV251	145	45	FWPV251 Serpin gene family protein	100	
ALPV2-343	278434-278772	FWPV251	145	112	FWPV251 Serpin gene family protein	100	
ALPV2-344	279121-278975			48			hypothetical protein, unique to ALPV2, containing a transmembrane helix
ALPV2-345	279847-279647	FWPV252	66	66	ORF FWPV252 hypothetical protein	100	
ALPV2-346	280214-279711	FWPV253	167	167	ORF FWPV253 C-type lectin gene family protein	100	
ALPV2-347	280296-280078	FWPV254	72	72	ORF FWPV254 hypothetical protein	100	
ALPV2-348	280727-280629			32			hypothetical protein, unique to ALPV2
ALPV2-349	281964-280708	FWPV255	418	418	ORF FWPV255 C4L/C10L-like gene family protein	100	
ALPV2-350	282306-282674	FWPV256	122	122	ORF FWPV256 EFc gene family protein	100	
ALPV2-351	282770-282465	FWPV257	101	101	ORF FWPV257 hypothetical protein	100	
ALPV2-352	283024-283395	FWPV258	123	123	ORF FWPV258 C-type lectin gene family protein	100	
ALPV2-353	283591-283451			46	MLPV323 hypothetical protein	56.8	
ALPV2-354	283842-283663			59	fpAU003 hypothetical protein	100	
**ALPV2-355**	**284528-283860**	**FWPV259**	**222**	**222**	**ORF FWPV259 conserved hypothetical protein**	**100**	
ALPV2-356	284725-284627			32			hypothetical protein, unique to ALPV2

ALPV2 Synteny	ALPV2 Genome Coordinates	FWPV Synteny	FWPV AA Size	ALPV2 AA Size	Best BLAST hits	ALPV2 AA Identity (%) Compared to Avipoxviruses	Notes
ALPV2-357	284977-284879			32			hypothetical protein, unique to ALPV2
ALPV2-358	285216-285088			42			hypothetical protein, unique to ALPV2
ALPV2-359	285404-286021	FWPV260	205	205	ORF FWPV260 C-type lectin gene family protein	100	

Note: ALPV2, albatrosspox virus 2; MLPV, mudlarkpox virus; MPPV, magpiepox virus; FGPV, flamingopox virus; PEPV, penguinpox virus; FeP2, pigeonpox virus; CNPV, canarypox virus. Truncated or fragmented ORFs of ALPV2 compared to FWPV are highlighted in blue text.
Bold and Italic: The 87 core genes conserved in all ChPVs which are involved in essential functions such as replication, transcription and virion assembly.
Bold: An additional 47 ORFs found to be uniquely conserved in the selected fully sequenced avian poxvirus genomes.
